# Development of an Aging-Related Gene Signature for Predicting Prognosis, Immunotherapy, and Chemotherapy Benefits in Rectal Cancer

**DOI:** 10.3389/fmolb.2021.775700

**Published:** 2022-01-10

**Authors:** Yangyang Wang, Yan Liu, Chunchao Zhu, Xinyu Zhang, Guodong Li

**Affiliations:** ^1^ Department of General Surgery, The Second Affiliated Hospital of Shandong First Medical University, Tai’an, China; ^2^ Department of Hematology, The Second Affiliated Hospital of Shandong First Medical University, Tai’an, China; ^3^ Department of Gastrointestinal Surgery, Renji Hospital Affiliated to Shanghai Jiaotong University School of Medicine, Shanghai, China

**Keywords:** aging, rectal cancer, prognosis, immunotherapy, tumor microenvironment, chemotherapy

## Abstract

**Objective:** Aging is the major risk factor for human cancers, including rectal cancer. Targeting the aging process provides broad-spectrum protection against cancers. Here, we investigate the clinical implications of aging-related genes in rectal cancer.

**Methods:** Dysregulated aging-related genes were screened in rectal cancer from TCGA project. A LASSO prognostic model was conducted, and the predictive performance was evaluated and externally verified in the GEO data set. Associations of the model with tumor-infiltrating immune cells, immune and stromal score, HLA and immune checkpoints, and response to chemotherapeutic agents were analyzed across rectal cancer. Biological processes underlying the model were investigated through GSVA and GSEA methods. Doxorubicin (DOX)-induced or replicative senescent stromal cells were constructed, and AGTR1 was silenced in HUVECs. After coculture with conditioned medium of HUVECs, rectal cancer cell growth and invasion were investigated.

**Results:** An aging-related model was established, consisting of KL, BRCA1, CLU, and AGTR1, which can stratify high- and low-risk patients in terms of overall survival, disease-free survival, and progression-free interval. ROC and Cox regression analyses confirmed that the model was a robust and independent predictor. Furthermore, it was in relation to tumor immunity and stromal activation as well as predicted the responses to gemcitabine and sunitinib. AGTR1 knockdown ameliorated stromal cell senescence and suppressed senescent stromal cell-triggered rectal cancer progression.

**Conclusion:** Our findings suggest that the aging-related gene signature was in relation to tumor immunity and stromal activation in rectal cancer, which might predict survival outcomes and immuno- and chemotherapy benefits.

## Introduction

Rectal cancer represents a common malignancy of the gastrointestinal tract that occurs in the lower part of the colon (Zhang et al., 2014). Approximately 15%–20% of patients with locally advanced rectal cancer have distant relapse despite all the therapeutic advances (Meng et al., 2020). Whether diagnosed as locally advanced or lymph node positive at any stage, the standard-of-care treatment is neoadjuvant chemotherapy as well as concomitant radiotherapy, followed by an operation (Tato-Costa et al., 2016). About 9%–20% of patients with locally advanced rectal cancer exhibit a pathological complete response to neoadjuvant radiochemotherapy, but 20%–40% of patients have almost no response (Kamran et al., 2019). Rectal cancer has the features of heterogeneous molecular subtypes and a variable clinical course as well as clinical outcomes (Sirinukunwattana et al., 2021). Molecular stratification is critical for forming homogeneous subgroups for personalized therapy and prognosis.

Aging presents the characteristics of gradual loss of physiological integrity, inducing damaged functions as well as increased risk of death (López-Otín et al., 2013). The deterioration acts as the main risk factor for human diseases, especially cancer (Calcinotto et al., 2019). Aging hallmarks are primarily classified into three types: 1) primary or the causes of aging-related injury, 2) antagonistic or the response to the injury, and 3) integrative or the results of the response as well as culprits of the aging phenotypes (McHugh and Gil, 2018). Identifying aging hallmarks may assist in conceptualizing aging studies as well as hinting at the tantalizing prospect for delaying aging-relevant malignancies through targeting the aging processes. It is of importance to identify pharmaceutical targets against cancer during aging with minimal side effects. Aging-related genes exert important roles in modulating cellular senescence, not only inhibiting tumors through regulating tumor cell senescence, but also promoting progression and undesirable prognosis of tumors (Yang et al., 2020). Nevertheless, there is a lack of evidence about the relationships of aging-related genes with the prognosis and immunity of rectal cancer. The Human Ageing Genomic Resources (HAGR) project provides the robust set of aging-specific genes through integrated analyses of the biology and genetics of aging process (Tacutu et al., 2018). This study evaluated the prognostic implication of aging-related genes and their associations with the tumor microenvironment in rectal cancer.

## Materials and Methods

### Data Acquisition and Preprocessing

Public gene-expression data as well as complete clinical annotation were searched from the Cancer Genome Atlas (TCGA; https://tcga-data.nci.nih.gov/tcga) and the Gene Expression Omnibus (GEO; https://www.ncbi.nlm.nih.gov/gds/) repository. Inclusion criteria were as follows: 1) histologically diagnosed with rectal cancer, 2) available RNA expression profiles, 3) complete follow-up information. Patients who had inactive follow-up were excluded. RNA sequencing (FPKM value) and survival data of 160 rectal cancer patients were curated from TCGA with the Genomic Data Commons website (https://portal.gdc.cancer.gov/repository) utilizing the TCGAbiolinks package on July 29, 2021 (Colaprico et al., 2016), as the discovery set. FPKM value was transformed into TPM format. Meanwhile, 342 rectal cancer patients were included from the GSE87211 data set (Hu et al., 2018) as the testing set. For the microarray profiling on the Agilent platform, the normalized matrix file was downloaded from the GEO repository, which was compatible with the TCGA data set. To ensure the two data sets as normal a distribution as possible as well as the compatibility of the data, the TPM-converted RNA-seq data and the microarray data that were preprocessed by RMA were both converted by log2 and then normalized using the scale method in the limma package. The clinical characteristics of rectal cancer patients in the TCGA and GSE87211 data sets are separately listed in Supplementary Tables S1, S2. In total, 307 aging-related genes were retrieved from the HAGR (https://genomics.senescence.info/), listed in Supplementary Table S3.

### Differential Expression Analysis and Functional Enrichment Analysis

Differential expression analysis of aging-related genes was conducted through comparison of normal and rectal cancer specimens utilizing the limma package (version 3.44.1) (Ritchie et al., 2015). The cutoff values of differentially expressed genes (DEGs) were as follows: |fold change (FC)| >1.5 and false discovery rate (FDR) <0.01. *P*-value was corrected with the Benjamini & Hochberg (BH) method. A heat map was depicted with the pheatmap package (version 1.12.0). Gene Ontology (GO) and Kyoto Encyclopedia of Genes and Genomes (KEGG) enrichment analysis of DEGs was carried out for revealing the biological functions and pathways utilizing clusterProfiler (version 3.17.0) and enrichplot packages (version 1.8.1) (Yu et al., 2012).

### Establishment of a Least Absolute Shrinkage and Selection Operator (LASSO) Cox Regression Model

For minimizing the risk of overfitting, the LASSO Cox regression model was employed for constructing a prognostic model based on dysregulated aging-related genes. The LASSO algorithm was utilized for variable selection as well as shrinkage *via* the glmnet package (Engebretsen and Bohlin, 2019). The penalty parameter (λ) that corresponds to the minimum partial likelihood deviance was identified through tenfold cross-verification in line with the minimum criteria. The risk score of each patient was calculated on the basis of the normalized expression of variables and their regression coefficients. The optimal cutoff value was determined utilizing survminer and survival packages as well as patients being separated into high- and low-risk groups. Kaplan–Meier curves of overall survival (OS), disease-free survival (DFS), and progression-free interval (PFI) analyses were presented between two groups in the discovery set, which were compared *via* log-rank tests. Time-dependent receiver operator characteristic (ROC) curves were conducted for evaluating predictive power of the prognostic model utilizing the survivalROC package (version 1.0.3). The testing set was employed for externally verifying the performance of the model in prediction of survival outcomes. The C-index of the aging-related gene model was calculated and compared with the existing gen models including Chen et al. signature (Chen et al., 2021) and Huang et al. signature (Huang et al., 2021).

### Uni- and Multivariate Cox Regression Analysis

Through univariate Cox regression analysis, the associations of age, gender, stage, T, N, M, and risk score with rectal cancer prognosis were analyzed in the discovery set. The predictive independency of these variables was estimated with multivariate Cox regression analysis. Forest plots were conducted for showing hazard ratio (HR), 95% confidence interval (CI), and *p*-value utilizing forestplot package (version 2.0.0).

### Nomogram Construction

Independent prognostic factors were included for building nomograms using the rms package (version 6.2–0) in the discovery set. Calibration curves were built for comparison of the actual with the nomogram-predicted 1-, 3-, and 5-year survival. Additionally, the C-index of the prognostic nomogram was calculated for evaluating the predictive efficacy.

### Estimation of Tumor Microenvironment Cell Infiltration

The single-sample gene-set enrichment analysis (ssGSEA) algorithm was applied for quantifying the abundance of immune cells across rectal cancer specimens (Hänzelmann et al., 2013). The gene set for marker genes was curated from a study of Charoentong et al. (Barbie et al., 2009; Charoentong et al., 2017). The enrichment score was calculated for representing the relative abundance of each tumor-infiltrating cell.

### Quantification of Immune Response Predictors

Utilizing the Estimation of Stromal and Immune Cells in Malignant Tumors using Expression Data (ESTIMATE) algorithm (Yoshihara et al., 2013), immune and stromal scores were calculated for predicting the infiltration levels of immune and stromal cells as well as tumor purity. The expression of human leukocyte antigen (HLA) and immune checkpoints were determined in each specimen.

### Estimation of Known Biological Processes

The gene sets of CD8 T effector, DNA damage repair, Pan-F-TBRS, antigen processing machinery, immune checkpoint, epithelial-mesenchymal transition (EMT) 1-3, FGFR3-related genes, KEGG discovered histones, angiogenesis, Fanconi anemia, cell cycle, DNA replication, nucleotide excision repair, homologous recombination, mismatch repair, WNT target, and cell cycle regulators were curated from previous studies (Rosenberg et al., 2016; Şenbabaoğlu et al., 2016; Mariathasan et al., 2018). The enrichment score of the above biological processes was estimated with ssGSEA method.

### Gene Set Enrichment Analysis

GSEA was presented through comparison of high- and low-risk samples (Subramanian et al., 2005). C2: curated gene sets, CP: canonical pathways and KEGG: KEGG gene sets were collected from the Molecular Signatures Database (version 7.4) as the reference set (Liberzon et al., 2015). The cutoff values were as follows: |nominal enrichment score (NES)| > 2 and FDR <0.05.

### Genetic Mutation and Methylation Analysis

Somatic copy-number alterations (CNAs) and methylation levels of BRCA1, CLU, and AGTR1 were retrieved from the cBioPortal (https://www.cbioportal.org/) (Gao et al., 2013). Associations of BRCA1, CLU, and AGTR1 expression with CNA values or methylation levels were estimated in rectal cancer with Spearman correlation analysis.

### Estimation of Chemotherapy Drug Response

By the Genomics of Drug Sensitivity in Cancer (GDSC) project (www.cancerRxgene.org) (Yang et al., 2013), the IC50 values of chemotherapy drugs (gemcitabine and sunitinib) were collected and determined with the pRRophetic package (Geeleher et al., 2014).

### Screening Small Molecule Drugs

DEGs between high- and low-risk groups were screened under the cutoff values of |FC| > 1.5 and FDR <0.05. The gene sets of upregulated and downregulated tags were separately uploaded onto the Connectivity map (CMap; http://portals.broadinstitute.org/cmap/) project (Lamb et al., 2006). Candidate small molecular agents were screened in line with |enrichment| >0.8 as well as permutation *p* < 0.05. CMap mode-of-action (MoA) analysis was utilized for exploring shared mechanisms of action among small molecular agents.

### Cell Culture

Human umbilical vein endothelial cells (HUVECs; American Type Culture Collection) were grown in RPMI 1640 medium (Gibco, United States) containing 10% fetal bovine serum (FBS; Gibco, United States) as well as antibiotics (1% penicillin–streptomycin). Human rectal cancer cell lines SW837 and SW1463 (American Type Culture Collection, United States) were maintained in Dulbecco’s modified Eagle medium (DMEM) plus 10% FBS as well as 1% penicillin–streptomycin. All cells were cultured at 37°C in an incubator with 5% CO_2_.

### Inducing Cell Senescence

For inducing replicative senescence, HUVECs were passaged when morphological signs of senescence appeared as growth stopped. For doxorubicin (DOX; Sigma, United States)-induced senescence, HUVECs were maintained with 50 nM DOX in RPMI 1640 medium lasting 72 h. Afterward, HUVECs were washed by PBS as well as left lasting 3 days before usage. Parental HUVECs with population doubling levels (PDL) 40–50 <20% senescent cells were utilized as a no-senescent control.

### Transfection and Conditioned Medium Collection

AGTR1 small interfering (siAGTR1) was transfected into HUVECs utilizing Lipofectamine^®^ 2000 transfection reagent (Invitrogen, United States). HUVECs were seeded onto a six-well plate (1×10^5^ cells/well) as well as maintained at 37°C lasting 24 h. Afterward, 1.5 ml medium that did not contain serum was added to each well with a mixture of 100 pmol siAGTR1 and Lipofectamine^®^ 2000 to incubate lasting 4 h at 37°C. The siRNA sequence is as follows: siAGTR1#1: 5′-GCA​GTA​GCC​AGC​AAT​TTG​A-3′, siAGTR1#2: 5′-ATA​AGA​AGG​TTC​AGA​TCC​A-3’. Following 48 h of transfection, HUVECs were harvested. For preparing the conditioned medium, senescent cells, nonsenescent cells, or cells with siAGTR1 pretransfection were incubated with medium lasting 72 h. The culture medium was collected as well as centrifuged, lasting 5 min. The supernatant was obtained and stored at −80°C prior to usage. SW837 and SW1463 cells were seeded onto a 96-well plate (3 × 10^3^ cells/well). Afterward, the cells were treated with conditioned medium lasting 48 h.

### Real-Time Quantitative Polymerase Chain Reaction (RT-qPCR)

Total RNA was extracted from HUVECs with TRIzol^®^ reagent (Invitrogen, United States), followed by being reverse transcribed into cDNA. Afterward, qPCR was carried out utilizing SYBR-Green Master Mix (Invitrogen, United States) as well as 7500 Fast RT-PCR system (Invitrogen, United States). The following primers were used for qPCR: AGTR1: 5′-ATT​TAG​CAC​TGG​CTG​ACT​TAT​GC-3′, 5′-CAG​CGG​TAT​TCC​ATA​GCT​GTG-3’; p53: 5′-CAG​CAC​ATG​ACG​GAG​GTT​GT-3′, 5′-TCA​TCC​AAA​TAC​TCC​ACA​CGC-3’; p21: 5′-TGT​CCG​TCA​GAA​CCC​ATG​C-3′, 5′-AAA​GTC​GAA​GTT​CCA​TCG​CTC-3’; GAPDH: 5′-ACA​ACT​TTG​GTA​TCG​TGG​AAG​G-3′, 5′-GCC​ATC​ACG​CCA​CAG​TTT​C-3’. The mRNA expression was quantified with 2^−∆∆Cq^ method as well as normalized by an internal reference gene GAPDH.

### Western Blot

Total protein was extracted from HUVECs with RIPA lysis buffer (Thermo Fisher Scientific, United States), which was quantified utilizing a BCA kit. Then, 40 µg protein was loaded and separated through 12% SDS-PAGE as well as transferred onto PVDF membrane (Millipore, United States). The membrane was blocked by 5% bovine serum albumin lasting 1 h at room temperature. Afterward, the membrane was incubated by AGTR1 (1/2000; ab124734; Abcam, United States), p53 (1/10,000; ab32389; Abcam, United States), p21 (1/1,000; ab188224; Abcam, United States) and GAPDH (1/2000; ab8245; Abcam, United States) overnight at 4°C as well as horseradish peroxide-conjugated secondary antibody (1/2000; ab7090; Abcam, United States) lasting 2 h at room temperature. A protein band was visualized with enhanced chemiluminescence reagent (Beyotime, China). Protein expression was quantified with ImageJ software with GAPDH as a loading control.

### Colony Formation Assay

SW837 and SW1463 cells were seeded in a six-well plate (1,000 cells/well). Under incubation at 37°C lasting 2 weeks, cells were fixed by 4% paraformaldehyde at room temperature lasting 15 min as well as stained by 0.5% crystal violet at room temperature lasting 10 min. Colonies with >50 cells were calculated utilizing a light microscope.

### Wound-Healing Assay

SW837 and SW1463 cells were seeded in a six-well plate (1×10^5^/well). When cells were confluent, a monolayer was scraped off utilizing a sterile pipette tip. Wound distance was investigated at 0 and 48 h with a light microscope (magnification, ×200).

### Transwell Assay

Transwell chambers (8 µM) coated with Matrigel (BD, United States) were applied for invasion detection. SW837 and SW1463 cells were seeded onto the upper chamber with serum-free medium (1×10^4^/well). Meanwhile, DMEM plus 10% FBS was added to the bottom chamber. After culturing lasting 24 h, invaded cells were fixed by 95% ethanol lasting 15 min at room temperature. Crystal violet solution was added to each chamber lasting 10 min at room temperature. The cells were counted under five random fields utilizing a light microscope (magnification, ×200).

### Statistical Analysis

All statistical analysis was achieved with R language (version 3.6.2) and SPSS software (version 23.0). Data are expressed as the mean ± standard deviation. Two-tailed Student’s *t*-test was utilized for evaluating the difference between two groups. One-way ANOVA followed by Dunnett’s test was applied for evaluating the difference between multiple groups. *p* < .05 was regarded as statistical significance.

## Results

### Expression Pattern and Biological Function of Aging-Related Genes in Rectal Cancer

**FIGURE 1:**
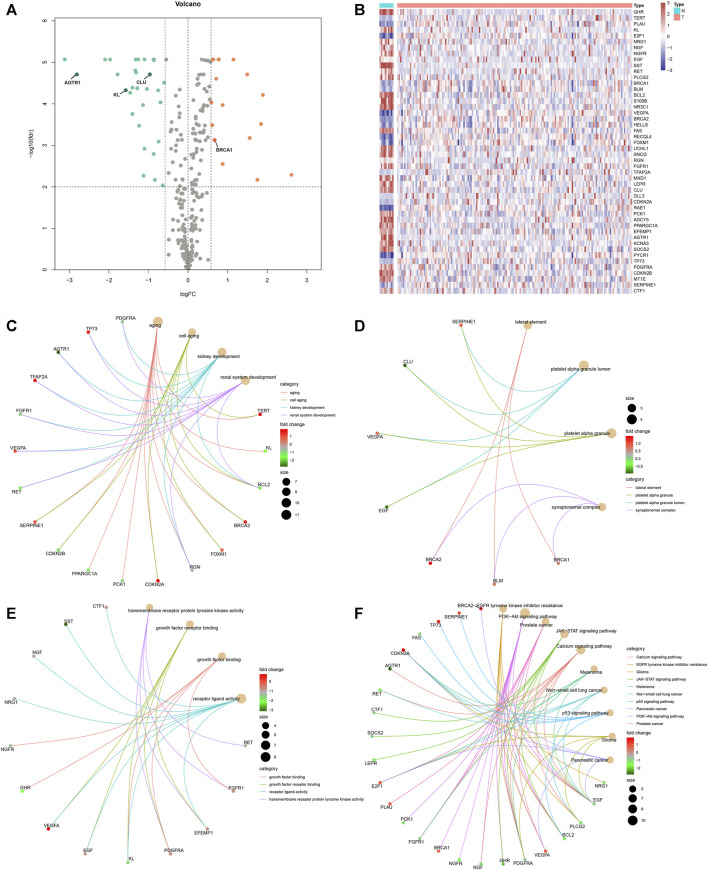
Expression pattern and biological function of aging-related genes in rectal cancer. Differentially expressed aging-related genes were identified between rectal cancer and normal specimens in the TCGA cohort with the cutoff values of |FC| > 1.5 and FDR <0.01. **(A, B)** Volcano and heat map visualized upregulated and downregulated aging-related genes in rectal cancer (T) than normal (N) specimens. Red means upregulated genes, blue means downregulated genes, and gray means nonsignificant genes. **(C–E)** Biological processes, molecular functions, and cellular components of dysregulated aging-related genes. **(F)** KEGG pathways enriched by dysregulated aging-related genes.

Under the cutoff values of |FC| > 1.5 and FDR <0.01, our study identified that 17 aging-related genes displayed markedly high expression and 31 displayed markedly lower expression in rectal cancer than normal tissues in the TCGA cohort (Table 1 and Figures 1A,B). Functional enrichment analysis was conducted to uncover the biological functions and pathways involving the dysregulated aging-related genes. In Figure 1C, aging-related genes were prominently enriched in biological processes of aging, cell aging, kidney development, and renal system development. Cellular components of lateral element, platelet alpha granule, platelet alpha granule lumen, and synaptonemal complex were markedly modulated by these aging-related genes (Figure 1D). The above genes possess the molecular functions of growth factor binding, growth factor receptor binding, receptor ligand activity, and transmembrane receptor protein tyrosine kinase activity (Figure 1E). In Figure 1F, carcinogenic pathways, including calcium signaling pathway, EGFR tyrosine kinase inhibitor resistance, glioma, JAK-STAT signaling pathway, melanoma, non-small cell lung cancer, p53 signaling pathway, pancreatic cancer, the PI3K-Akt signaling pathway, and prostate cancer were markedly enriched by the above genes. Collectively, these abnormally expressed aging-related genes may exert critical roles in aging and carcinogenesis.

**TABLE 1:** Differentially expressed aging-related genes in rectal cancer.

Gene	Normal mean	Tumor mean	logFC	P-value	FDR
SST	5.291386	0.611638	−3.1129	1.21E-07	8.55E-06
GHR	2.261302	0.529536	−2.09435	2.89E-07	8.55E-06
ADCY5	2.28577	0.583306	−1.97036	4.93E-07	8.55E-06
NR3C1	2.92778	1.17025	−1.32299	3.66E-07	8.55E-06
BCL2	2.18458	0.913904	−1.25724	3.91E-07	8.55E-06
CDKN2B	4.346567	2.027179	−1.1004	3.09E-07	8.55E-06
FAS	3.744046	2.046475	−0.87146	4.77E-07	8.55E-06
MT1E	7.348482	4.050582	−0.85932	2.70E-07	8.55E-06
RAE1	2.431015	3.743455	0.622812	1.46E-07	8.55E-06
PYCR1	3.510742	5.467207	0.639028	2.89E-07	8.55E-06
VEGFA	2.217516	3.777256	0.768394	4.04E-07	8.55E-06
E2F1	2.285757	3.923648	0.779524	3.31E-07	8.55E-06
RECQL4	1.624488	3.596862	1.146754	4.18E-07	8.55E-06
SOCS2	2.275753	1.251213	−0.86302	5.82E-07	8.90E-06
PPARGC1A	2.189429	0.87022	−1.3311	1.27E-06	1.57E-05
SNCG	2.906813	1.168448	−1.31484	1.49E-06	1.76E-05
AGTR1	1.907258	0.272117	−2.8092	2.11E-06	1.96E-05
KCNA3	0.85956	0.25018	−1.78063	1.80E-06	1.96E-05
CLU	5.685867	2.911547	−0.96559	2.11E-06	1.96E-05
BRCA2	0.428297	1.205366	1.492786	2.11E-06	1.96E-05
PLAU	2.648905	4.337372	0.711425	2.88E-06	2.49E-05
MXD1	4.794588	3.154305	−0.60408	3.80E-06	3.08E-05
UCHL1	3.310661	1.250929	−1.40412	5.81E-06	4.08E-05
LEPR	1.517166	0.624065	−1.28161	6.17E-06	4.22E-05
PCK1	5.397511	2.627154	−1.03879	6.75E-06	4.38E-05
KL	1.060475	0.356252	−1.57374	7.60E-06	4.71E-05
EFEMP1	4.677153	2.769171	−0.75618	7.83E-06	4.73E-05
S100B	3.526386	1.280192	−1.46183	9.35E-06	5.40E-05
CDKN2A	0.372944	1.381823	1.889541	1.08E-05	6.12E-05
PDGFRA	3.155919	1.946584	−0.69712	1.77E-05	9.21E-05
FOXM1	2.499946	3.763778	0.590,285	1.82E-05	9.29E-05
PLCG2	1.558625	0.708668	−1.13709	2.22E-05	0.000107
HELLS	0.935051	1.710483	0.871287	2.16E-05	0.000107
NGFR	1.641489	0.619482	−1.40587	4.11E-05	0.000175
TERT	0.17766	0.63769	1.843736	7.85E-05	0.000305
BRCA1	1.308927	1.99851	0.61054	8.50E-05	0.000325
RET	1.060753	0.450442	−1.23567	9.20E-05	0.000337
TP73	0.229904	0.676393	1.556833	0.000215	0.000665
BLM	0.995986	1.584562	0.669886	0.000256	0.000747
FGFR1	2.800322	1.673316	−0.74288	0.000296	0.000811
NRG1	0.699304	0.326287	−1.09978	0.000458	0.001204
EGF	0.626976	0.343338	−0.86878	0.000637	0.001609
SERPINE1	1.801445	3.299017	0.872881	0.001207	0.002828
DLL3	0.052596	0.32155	2.61203	0.00229	0.005133
NGF	0.769865	0.353042	−1.12477	0.002414	0.005365
TFAP2A	0.152011	0.511589	1.750806	0.003165	0.006745
CTF1	1.599103	0.895457	−0.83657	0.00323	0.006774
RGN	0.861205	0.552279	−0.64096	0.004647	0.009223

### Establishment and External Verification of an Aging-Related Gene Model for Rectal Cancer Prognosis

**FIGURE 2:**
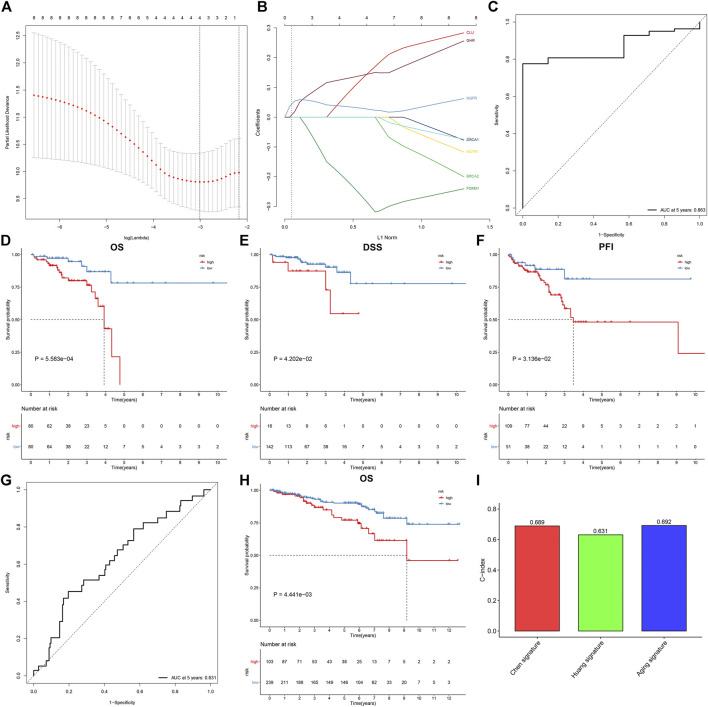
Establishment and external verification of an aging-related gene model for rectal cancer prognosis. **(A)** Partial likelihood deviance corresponding to different lambda values. **(B)** LASSO coefficient profiles. **(C)** ROC curves at 5-year survival in the discovery set. **(D–F)** Kaplan–Meier curves of OS, DSS, and PFI in high- and low-risk patients in the discovery set. **(G, H)** ROC curves at 5-year survival as well as OS analysis for rectal cancer patients in the testing set. **(I)** Comparison of the C-index among the aging-related gene model and existing gene models (Chen et al. signature and Huang et al. signature).

Through the LASSO Cox regression method, we developed a prognostic model on the basis of the expression profiling of aging-related genes in the discovery set. This model contained KL, BRCA1, CLU, and AGTR1 (Figures 2A,B). The risk score was determined in line with the formula: risk score = BRCA1 expression * (-0.230004762718913) + CLU expression * 0.0820122510344611 + AGTR1 expression * 0.0991120882567845. The ROC curve was conducted for investigating the predictive efficacy of the model. In Figure 2C, the area under the curve (AUC) of 5-year survival was 0.863, indicative of the excellent performance in prediction of prognosis. In line with the optimal value, rectal cancer patients were stratified into high- and low-risk subgroups. Survival analysis uncovered high-risk patients presented poorer OS, DSS, and PFI than low-risk patients (Figures 2D–F). We further externally validated the gene model in the testing set. The AUC at 5 years was 0.631, confirming the favorable predictive performance (Figure 2G). Consistently, high-risk patients were indicative of worse OS (Figure 2H). Compared with existing gene models (Chen et al. signature and Huang et al. signature), the aging-related gene model had higher C-index, indicating that this model presented higher efficacy in predicting rectal cancer prognosis (Figure 2I).

### Aging-Related Gene Model as an Independent Risk Factor of Rectal Cancer

**FIGURE 3:**
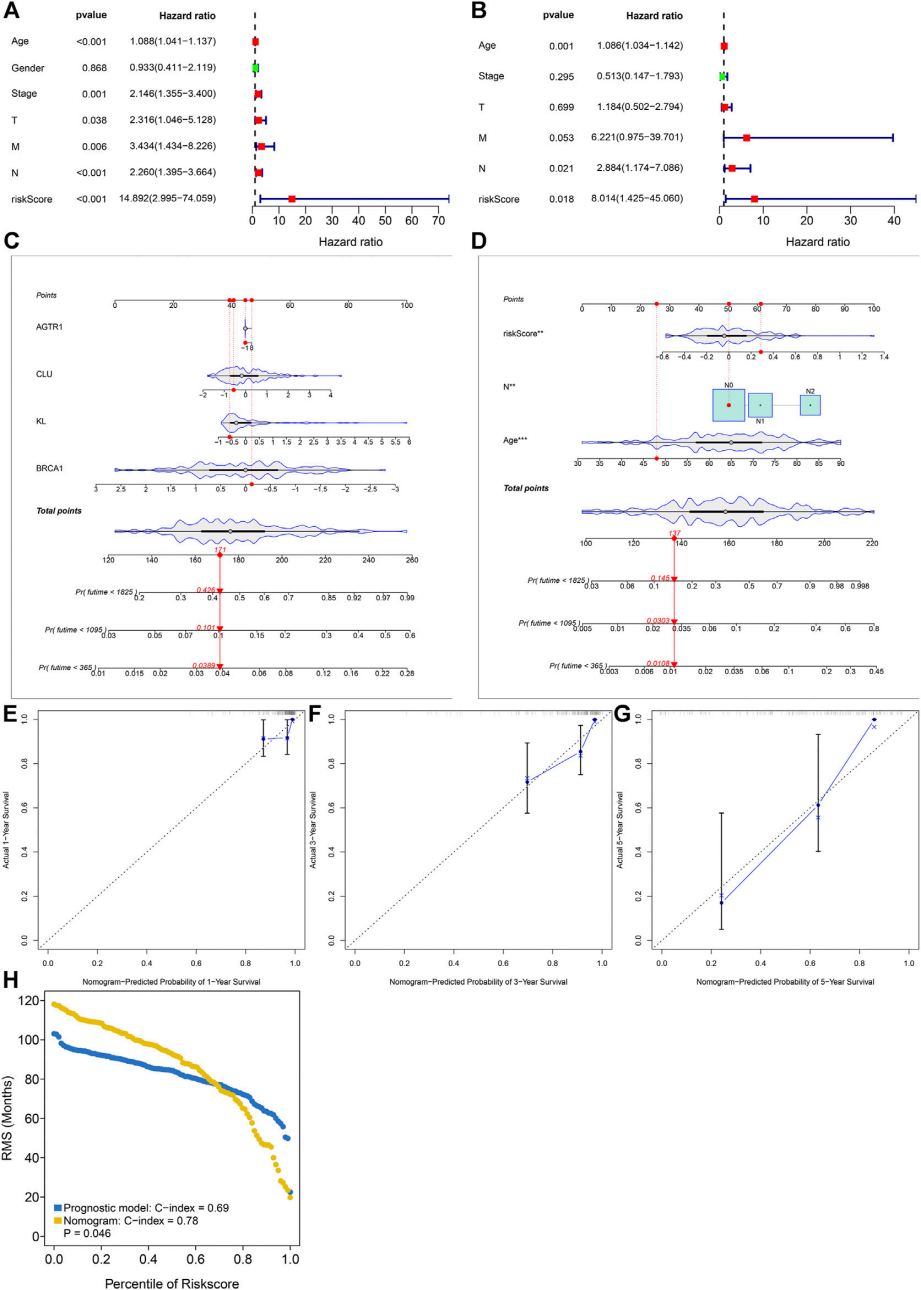
Aging-related gene model as an independent risk factor of rectal cancer in the discovery set. **(A, B)** Uni- and multivariate Cox regression models for investigating the associations of the aging-related gene model and conventional clinical factors with rectal cancer prognosis. **(C)** The prognostic nomogram containing KL, BRCA1, CLU, and AGTR1. **(D)** The prognostic nomogram that includes independent risk factors. **(E–G)** Calibration curves for the associations between actual and nomogram-estimated probabilities of 1-, 3- and 5-year survival. **(H)** The C-index values of the aging-related gene model and the prognostic nomogram.

Through the univariate Cox regression model, age, stage, T, N, M, and aging-related gene model were markedly correlated to rectal cancer prognosis (Figure 3A). Following multivariate Cox regression, age, N, and aging-related gene model acted as independent risk factors of rectal cancer (Figure 3B). The nomogram containing KL, BRCA1, CLU, and AGTR1 was conducted for prediction of 1-, 3-, and 5-year survival (Figure 3C). Moreover, the prognostic nomogram was developed through incorporating independent risk factors (age, N, and aging-related gene model) in Figure 3D. Calibration curves confirmed the high consistency between actual and the nomogram-estimated probabilities of 1-, 3-, and 5-year survival (Figures 3E–G). Additionally, the C-index values of the aging-related gene model and the prognostic nomogram were separately 0.69 and 0.78, indicating the good predictive potency in rectal cancer prognosis (Figure 3H).

### Association of Aging-Related Gene Signature With the Tumor Immune Microenvironment

**FIGURE 4:**
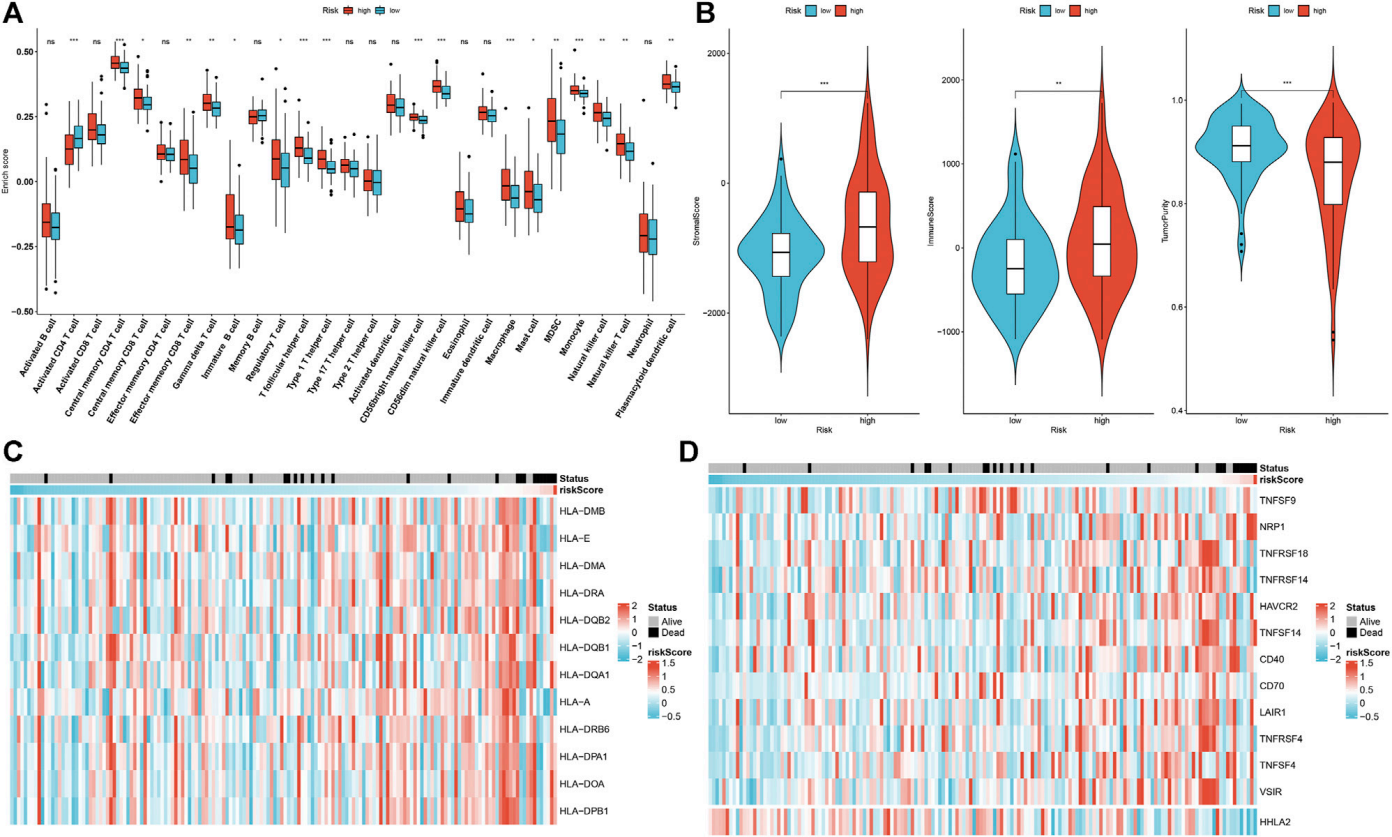
Association of aging-related gene signature with the tumor immune microenvironment of rectal cancer in the discovery set. **(A)** Comparison of the abundance of tumor-infiltrating lymphocytes in high- and low-risk subgroups. **(B)** Comparison of stromal score, immune score, and tumor purity in high- and low-risk subgroups. **(C)** Distribution of HLA gene expression in high- and low-risk specimens. **(D)** Distribution of immune checkpoint expression in high- and low-risk specimens. Ns: not significant; *p < .05; **p < .01; ***p < .001.

Through the ssGSEA method, we estimated the abundance of tumor-infiltrating lymphocytes. High-risk patients were characterized by increased infiltration of central memory CD4^+^ T cell, central memory CD8^+^ T cell, gamma delta T cell, immature B cell, regulatory T cell, T follicular helper cell, type 1 T helper cell, CD56bright natural killer cell, CD56dim natural killer cell, macrophage, mast cell, MDSC, monocyte, natural killer cell, natural killer T cell and plasmacytoid dendritic cell in the discovery set (Figure 4A). In Figure 4B, higher immune and stromal scores as well as reduced tumor purity were found in high-risk patients. Similar consequences were found in the testing set (Supplementary Figures S1A,B). Moreover, we observed that most of the HLA genes and immune checkpoints presented increased expression in high-risk patients (Figures 4C,D). The above findings indicate that the aging-related gene signature was in relation to tumor immunity.

### Association of Aging-Related Gene Signature With Stromal Activation

**FIGURE 5:**
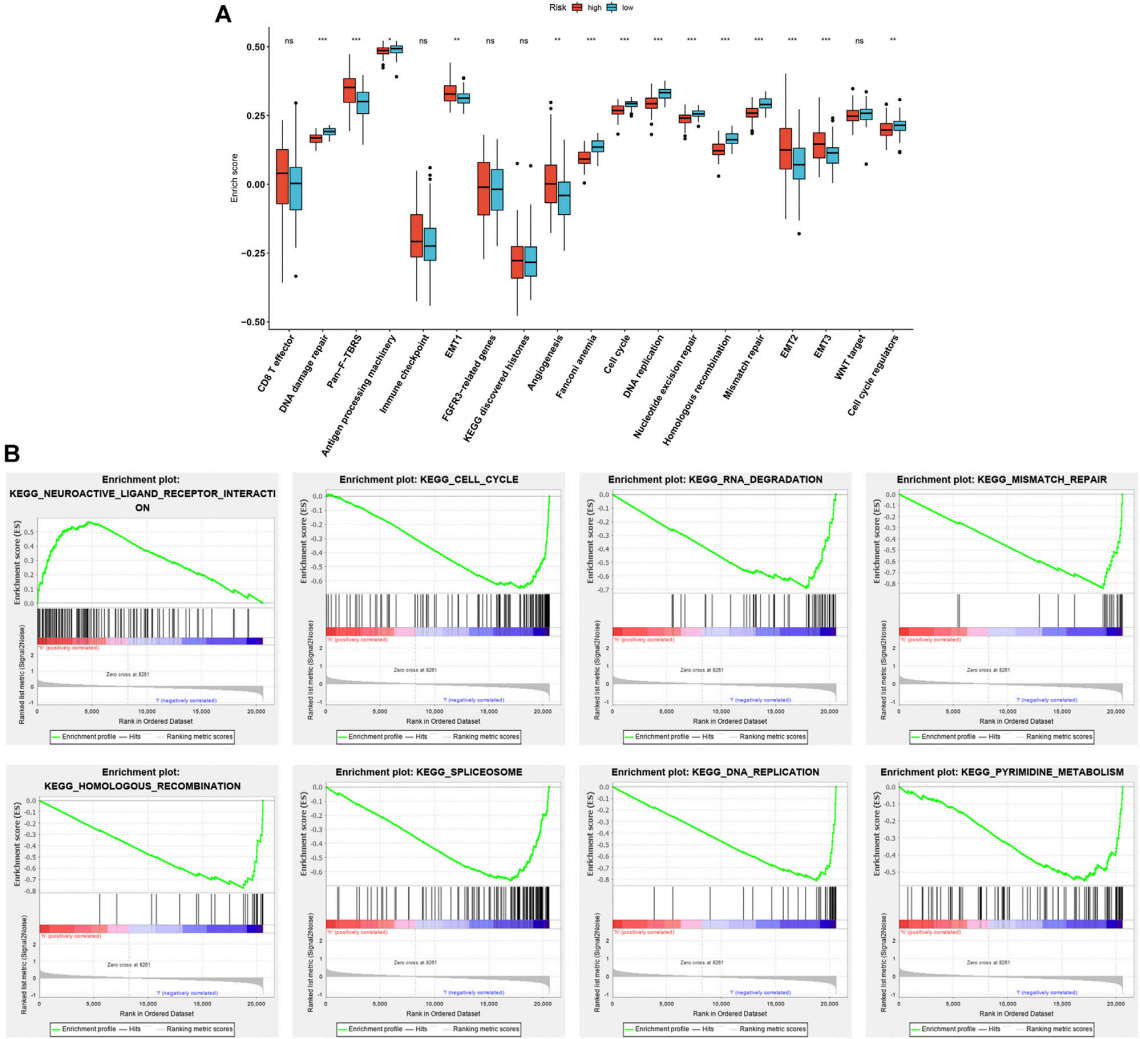
Association of aging-related gene signature with stromal activation in the discovery set. **(A)** Comparisons of the activation of known biological processes in high- and low-risk patients. **(B)** GSEA results for KEGG pathways associated with aging-related gene signature. Ns: not significant; *p < .05; **p < .01; ***p < .001.

As depicted in Figure 5A, high-risk patients presented the activation of pan-F-TBRS, EMT1-3, and angiogenesis. Meanwhile, low-risk patients displayed the activation of DNA damage repair, antigen processing machinery, Fanconi anemia, cell cycle, DNA replication, nucleotide excision repair, mismatch repair, and cell cycle regulators in the discovery set. This indicates that the aging-related gene signature was positively associated with stromal activation. Similar results were found in the testing set (Supplementary Figure S1C). GSEA results also confirmed that this gene signature was positively correlated to neuroactive ligand receptor interaction as well as negatively correlated to cell cycle, RNA degradation, mismatch repair, homologous recombination, spliceosome, DNA replication, and pyrimidine metabolism (Figure 5B).

### Aging-Related Gene Signature Chemotherapy Predicts Chemotherapy Benefit in Rectal Cancer

**FIGURE 6:**
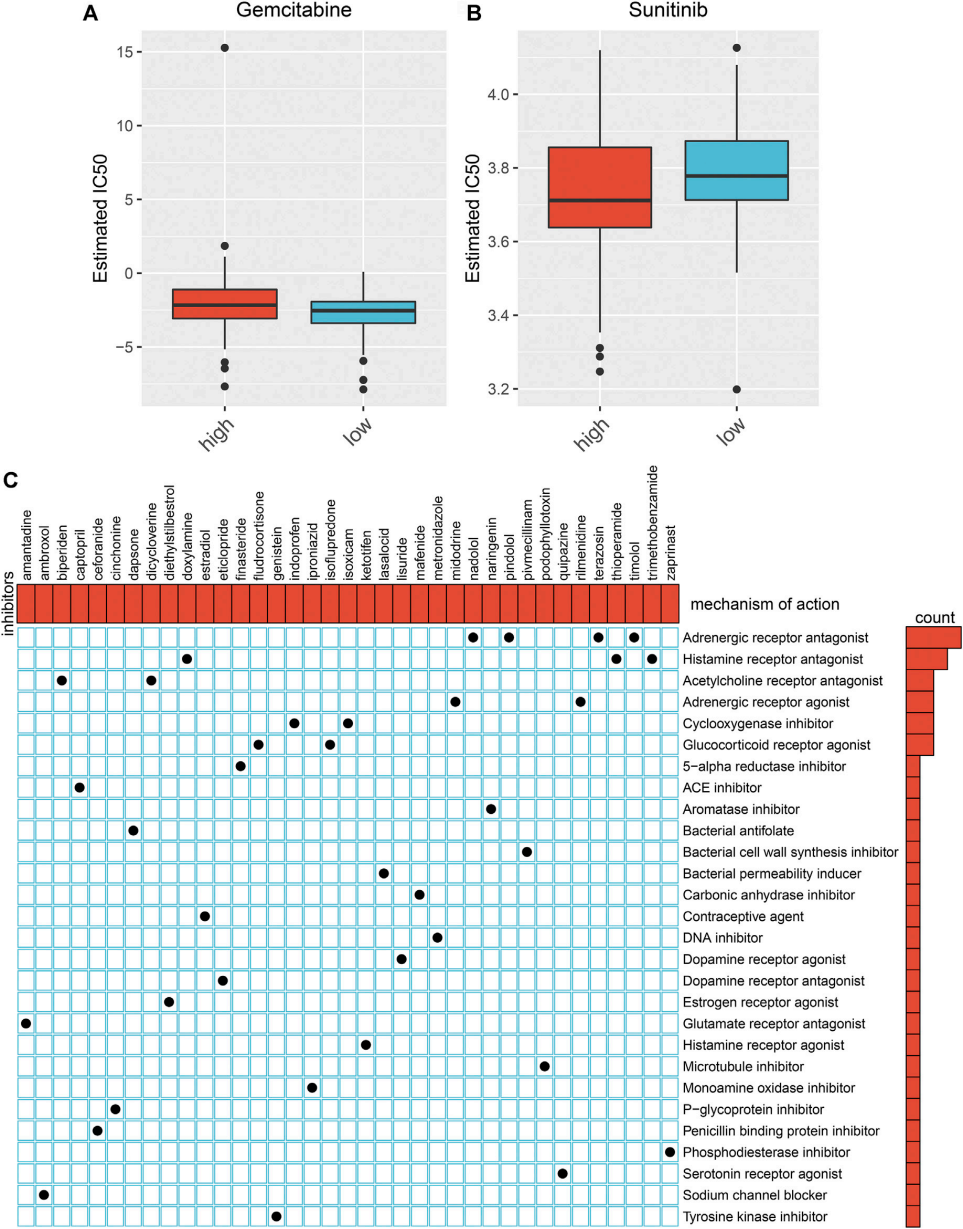
Prediction of chemotherapy benefit and small molecular agents based on the aging-related gene model. **(A, B)** Comparison of IC50 value of gemcitabine and sunitinib in high- and low-risk subgroups. **(C)** Shared molecular mechanisms among small molecular agents *via* MoA.

We estimated the IC50 value of gemcitabine and sunitinib in each rectal cancer. In comparison to the high-risk subgroup, there was a prominently reduced IC50 value of gemcitabine (*p* < .024) in the low-risk subgroup (Figure 6A). This indicates that low-risk patients displayed more benefit to gemcitabine. In contrast, a markedly lower IC50 value of sunitinib (*p* < 0.017) was found in the high-risk subgroup than the low-risk subgroup (Figure 6B), indicating that high-risk patients were more likely to benefit from sunitinib.

### Prediction of Small Molecular Agents Against Rectal Cancer

We identified 218 DEGs between the high- and low-risk subgroups (Supplementary Table S4). In total, 35 small molecular agents were identified against rectal cancer based on the above DEGs by CMap analysis with the cutoff values of |enrichment| > 0.8 and *p* < .05 (Table 2). Shared molecular mechanisms were predicted *via* MoA. As depicted in Figure 6C, nadolol, pindolol, terazosin, and timolol share an adrenergic receptor antagonist; doxylamine, thioperamide, and trimethobenzamide share a histamine receptor antagonist; biperiden and dicycloverine share an acetylcholine receptor antagonist; midodrine and rilmenidine share an adrenergic receptor agonist; indoprofen and isoxicam share a cyclooxygenase inhibitor; and fludrocortisone and isoflupredone share a glucocorticoid receptor agonist.

**TABLE 2:** Potential small molecular agents against rectal cancer by CMap analysis.

CMap name	Mean	n	Enrichment	P-value	Specificity	Percent non-null
menadione	0.897	2	0.992	0.00004	0.0089	100
GW-8510	0.842	4	0.954	0	0.0663	100
MS-275	0.783	2	0.939	0.00718	0.0996	100
quinostatin	0.778	2	0.922	0.01217	0.1437	100
8-azaguanine	0.841	4	0.92	0.00004	0.0118	100
trazodone	0.756	3	0.919	0.00112	0.0149	100
irinotecan	0.777	3	0.916	0.00118	0.1409	100
sanguinarine	0.825	2	0.914	0.01481	0.125	100
chrysin	0.775	3	0.904	0.00182	0.0088	100
0175029-0000	0.796	6	0.902	0	0.0177	100
piperlongumine	0.768	2	0.898	0.02163	0.1234	100
camptothecin	0.851	3	0.893	0.00238	0.183	100
rottlerin	0.719	3	0.887	0.00292	0.0933	100
5248896	0.727	2	0.883	0.02801	0.0375	100
DL-thiorphan	0.74	2	0.876	0.03074	0.1103	100
thioguanosine	0.835	4	0.865	0.00044	0.0177	100
phenoxybenzamine	0.777	4	0.863	0.00046	0.2277	100
apigenin	0.799	4	0.823	0.00167	0.0703	100
bepridil	0.692	4	0.816	0.00217	0.0389	100
alsterpaullone	0.759	3	0.814	0.01288	0.1337	100
azacitidine	0.739	3	0.811	0.01366	0.1058	100
isoflupredone	−0.787	3	−0.898	0.002	0.0833	100
Nadolol	−0.668	4	−0.877	0.00052	0	100
vigabatrin	−0.651	3	−0.877	0.00379	0.007	100
viomycin	−0.672	4	−0.876	0.00052	0.0432	100
adiphenine	−0.763	5	−0.867	0.00008	0.0323	100
Prestwick-1082	−0.652	3	−0.851	0.00655	0.0902	100
heptaminol	−0.666	5	−0.842	0.00026	0.0137	100
3-acetamidocoumarin	−0.678	4	−0.84	0.00117	0.0195	100
Prestwick-983	−0.626	3	−0.839	0.00837	0.0206	100
cinchonine	−0.596	4	−0.832	0.00145	0.0185	100
chenodeoxycholic acid	−0.638	4	−0.826	0.00177	0.0385	100
podophyllotoxin	−0.687	4	−0.822	0.00189	0.0588	100
trihexyphenidyl	−0.643	3	−0.807	0.0144	0.0376	100
atractyloside	−0.665	5	−0.806	0.00064	0.0076	100

### CNAs and Methylation Levels of BRCA1, CLU, AGTR1, and KL Across Rectal Cancer

**FIGURE 7:**
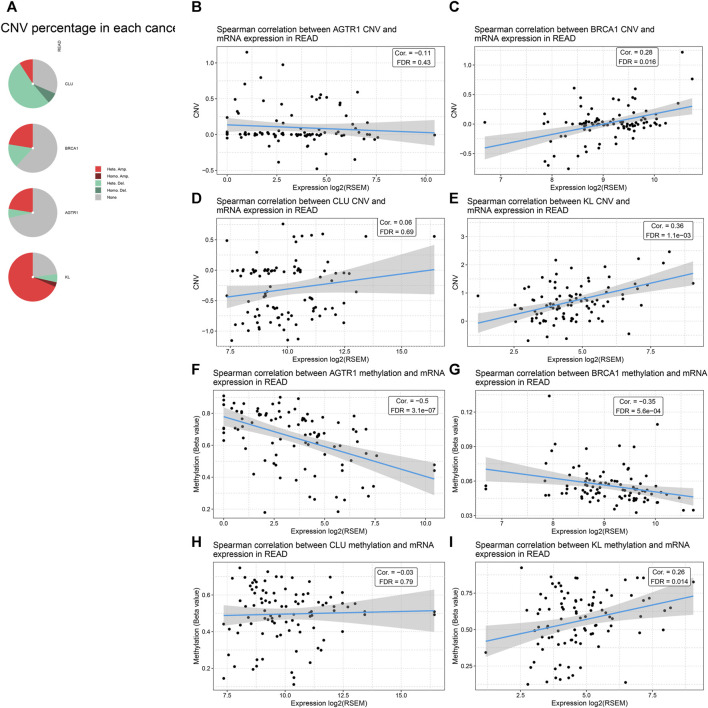
Analysis of CNAs and methylation levels of BRCA1, CLU, AGTR1, and KL across rectal cancer through cBioPortal. **(A)** Landscape of mutation types in BRCA1, CLU, AGTR1, and KL across rectal cancer. **(B–E)** Spearman correlation of BRCA1, CLU, AGTR1, and KL expression with their corresponding CNA values in rectal cancer. **(F–I)** Spearman correlation of BRCA1, CLU, AGTR1, and KL expression with their corresponding methylation levels in rectal cancer.

Through the cBioPortal tool, we evaluated CNAs of BRCA1, CLU, AGTR1, and KL across rectal cancer. As depicted in Figure 7A, CLU had widespread CNA deletion as well as BRCA1, AGTR1, and KL displaying the widespread CNA amplification. Through Spearman correlation analysis, the association of BRCA1, CLU, AGTR1, and KL expression with the CNA value was estimated across rectal cancer. As a result, AGTR1 expression was negatively correlated to its CNA (Figure 7B), whereas BRCA1, CLU, and KL expression was positively associated with CNAs (Figures 7C–E). This study also evaluated the correlation of BRCA1, CLU, AGTR1, and KL expression with methylation levels in rectal cancer. Our results demonstrate that AGTR1, BRCA1, and CLU expression possess the negative correlations to methylation (Figures 7F–H), whereas KL exhibits the positive correlation to methylation (Figure 7I).

### Association of BRCA1, CLU, AGTR1, and KL With Tumor-Infiltrating Immune Cells and Known Biological Processes

**FIGURE 8:**
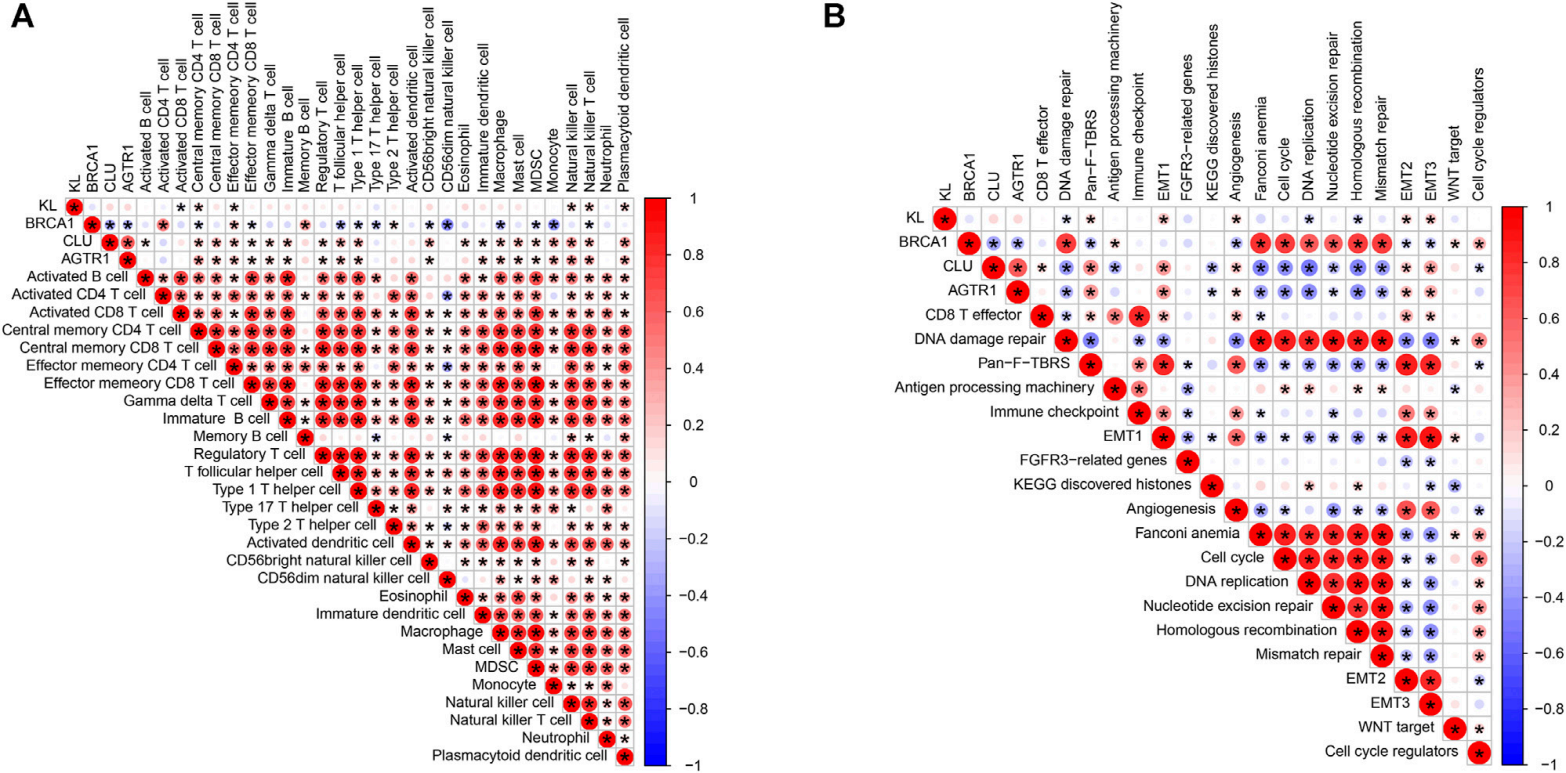
Association of BRCA1, CLU, AGTR1, and KL with tumor-infiltrating immune cells and known biological processes. **(A)** Spearman correlations of BRCA1, CLU, AGTR1, and KL with tumor-infiltrating immune cells across rectal cancer. **(B)** Spearman correlations of BRCA1, CLU, AGTR1, and KL with known biological processes across rectal cancer. The bigger the bubble, the stronger the correlation. Red means positive correlation, and blue means negative correlation.

In Figure 8A, KL displays positive correlations to central memory CD4^+^ T cell, effector memory CD4^+^ T cell, natural killer cell, natural killer T cell, and plasmacytoid dendritic cell while displaying a negative correlation to activated CD8^+^ T cell. BRCA1 possessed positive correlations to activated CD4^+^ T cell, effector memory CD4^+^ T cell, memory B cell, and type 2 T helper cell as well as having negative correlations to central memory CD4^+^ T cell, effector memory CD8^+^ T cell, T follicular helper cell, type 1 T helper cell, type 17 T helper cell, CD56bright natural killer cell, CD56dim natural killer cell, macrophage, MDSC, monocyte, and natural killer T cell. CLU was positively associated with activated B cell, central memory CD4^+^ T cell, central memory CD8^+^ T cell, effector memory CD4^+^ T cell, effector memory CD8^+^ T cell, gamma delta T cell, immature B cell, regulatory T cell, T follicular helper cell, type 1 T helper cell, CD56bright natural killer cell, eosinophil, immature dendritic cell, macrophage, mast cell, MDSC, natural killer cell, natural killer T cell, and plasmacytoid dendritic cell. Moreover, we observed that AGTR1 displayed positive correlations to central memory CD4^+^ T cell, central memory CD8^+^ T cell, effector memory CD4^+^ T cell, effector memory CD8^+^ T cell, gamma delta T cell, immature B cell, regulatory T cell, T follicular helper cell, type 1 T helper cell, CD56bright natural killer cell, immature dendritic cell, macrophage, mast cell, MDSC, natural killer cell, natural killer T cell and plasmacytoid dendritic cell. We also evaluated the correlations of BRCA1, CLU, and AGTR1 with known biological processes. As depicted in Figure 8B, KL was positively associated with pan-F-TBRS, EMT1-3, and angiogenesis but was negatively correlated to DNA damage repair, DNA replication, and homologous recombination. BRCA1 exhibited positive associations with DNA damage repair, antigen processing machinery, Fanconi anemia, cell cycle, DNA replication, nucleotide excision repair, homologous recombination, mismatch repair, WNT target and cell cycle regulators while exhibiting negative associations with pan-F-TBRS, angiogenesis, EMT2, and EMT3. CLU displayed positive correlations to CD8+T cell, pan-F-TBRS, EMT1-3, and angiogenesis. In contrast, CLU possessed negative associations with DNA damage repair, antigen-processing machinery, KEGG discovered histones, Fanconi anemia, cell cycle, DNA replication, nucleotide excision repair, homologous recombination, mismatch repair, and cell cycle regulators. AGTR1 had positive correlations to pan-F-TBRS, EMT1-3, and angiogenesis, whereas it had negative associations with DNA damage repair, KEGG-discovered histones, Fanconi anemia, cell cycle, DNA replication, nucleotide excision repair, homologous recombination, and mismatch repair.

### Silencing AGTR1 ameliorates Stromal Cell Senescence

**FIGURE 9:**
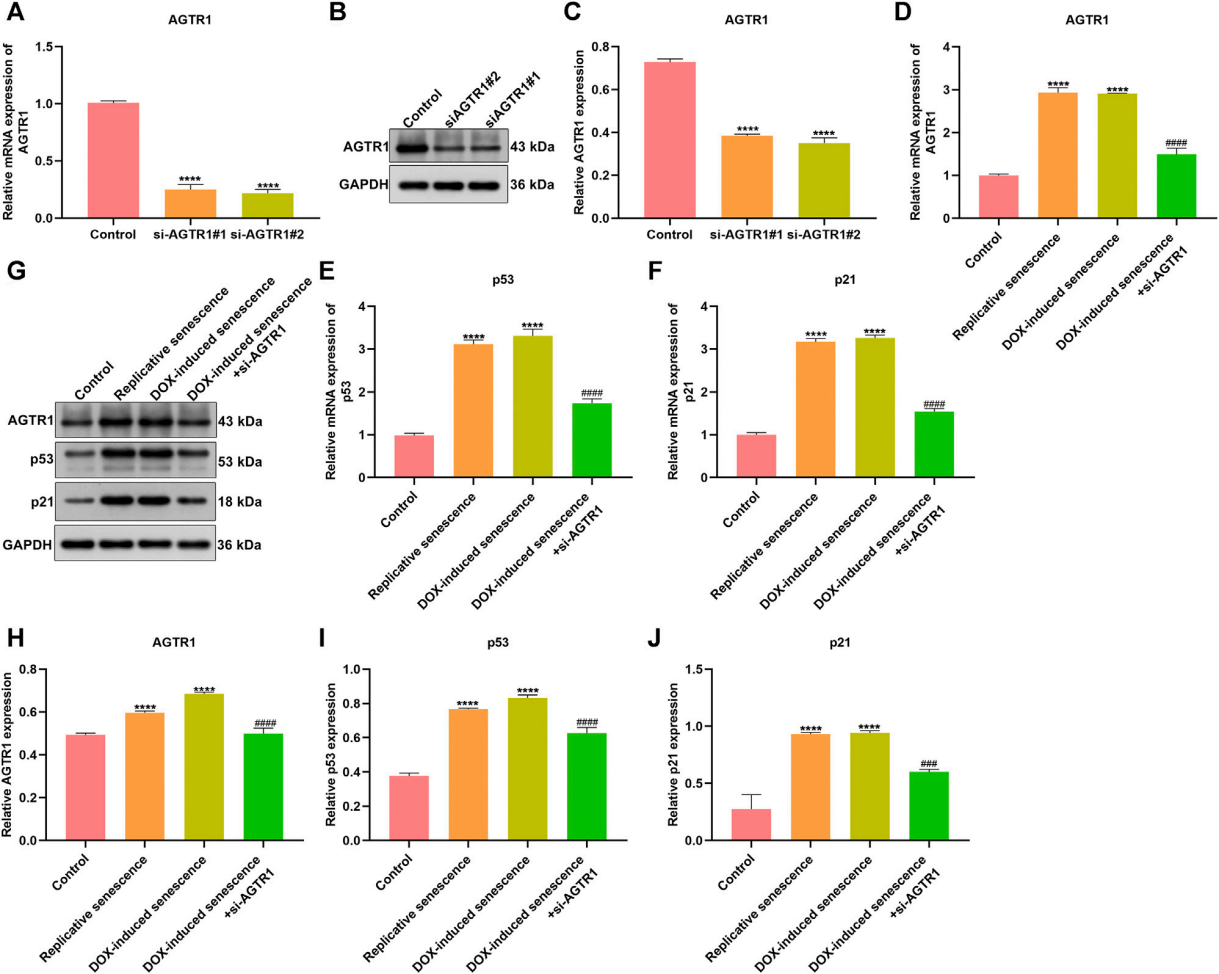
Silencing AGTR1 ameliorates stromal cell senescence. **(A–C)** RT-qPCR and Western blotting detected AGTR1 expression in HUVECs transfected by siAGTR1. **(D–F)** RT-qPCR and **(G–J)** Western blotting were utilized for examining AGTR1, p53, and p21 expression in four groups: control, replicative senescence, DOX-induced senescence, and DOX-induced senescence + siAGTR1 groups. Compared with control, ****p < .0001. Compared with DOX-induced senescence, ###*p* < .001; ####*p* < .0001.

We further focus on the role of AGTR1 on cellular senescence. AGTR1 expression was successfully silenced by its siRNAs in stromal cells HUVECs (Figures 9A–C). DOX-induced and replicative senescent HUVECs were conducted. We observed the increase in AGTR1 as well as cellular senescence markers p53 and p21 expression in senescent HUVECs (Figures 9D–J). In contrast, AGTR1 knockdown reduced p53 and p21 expression in senescent HUVECs.

### Silencing AGTR1 Suppresses Senescent Stromal Cell–Triggered Rectal Cancer Progression

**FIGURE 10:**
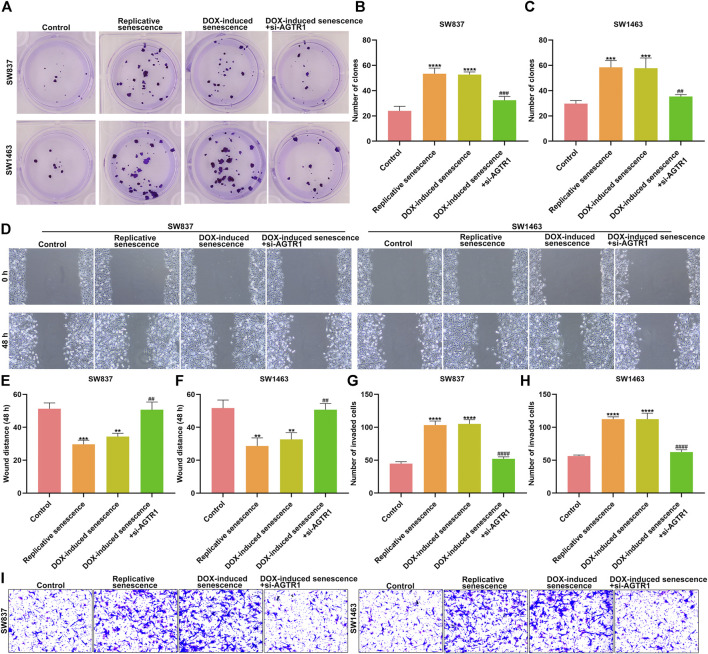
Silencing AGTR1 suppresses senescent stromal cell-triggered rectal cancer progression. **(A–C)** Colony formation assay was presented for investigating proliferation of SW837 and SW1483 rectal cancer cells co-cultured with the conditioned medium of senescent HUVECs with AGTR1 knockdown. **(D–F)** Wound healing assay was utilized for examining migration of SW837 and SW1483 cells co-cultured with the conditioned medium of senescent HUVECs with AGTR1 knockdown. **(G–I)** Transwell assay was presented for examining invasion of SW837 and SW1483 cells cocultured with the conditioned medium of senescent HUVECs with AGTR1 knockdown. Compared with control, **p < .01; ***p < .001; ****p < .0001. Compared with DOX-induced senescence, ##*p* < .01; ###*p* < .001; ####*p* < .0001.

For observing the effect of AGTR1 on senescent stromal cell–triggered rectal cancer progression, SW837 and SW1483 rectal cancer cells were cocultured with the conditioned medium of senescent HUVECs with AGTR1 knockdown. Our results show that replicative and DOX-induced senescent stromal cells prominently induced proliferation (Figures 10A–C), migration (Figures 10D–F) as well as invasion (Figures 10G–I) of SW837 and SW1483 cells, which were weakened by AGTR1 knockdown.

## Discussion

Aging acts as a dominant risk factor for cancer. This study conducted an aging-related genetic signature for rectal cancer, which might improve individualized therapy as well as offer promising novel molecular markers and predictors against immune- and chemotherapy.

Though the local relapse as well as OS duration has improved, distant relapse rates have not markedly reduced. Approximately 30% of patients receiving curative treatment ultimately experience distant metastasis (Serna et al., 2020). Herein, the aging-related gene model can accurately estimate high-risk rectal cancer patients for OS, DSS, and PFI. Different from conventional clinicopathological characteristics, this model acts as an independent risk factor of rectal cancer prognosis. Our univariate Cox regression analysis results show that T, N, and M stages were all risk factors of rectal cancer prognosis. However, after including other prognostic variables, only the N stage could independently predict patients’ prognosis, consistent with previous studies (Di et al., 2020; Zhu et al., 2021). Our data indicate that more reliable prognostic markers should be included in the clinic.

It is suggested that preoperative neoadjuvant chemotherapy is of considerable importance upon rectal cancer therapy, which improves the rate of curative resection as well as prominently decreases local relapse (Ji et al., 2018). Nevertheless, adjuvant chemotherapy against patients who receive preoperative chemoradiotherapy and operation remains controversial. This study indicates that the aging-related gene model might be utilized for prediction of the response to chemotherapeutic agents (gemcitabine and sunitinib). High-risk patients are more likely to benefit from sunitinib. In contrast, low-risk patients exhibit higher sensitivity to gemcitabine. Rectal cancer displays high infiltration levels of tumor-infiltrating lymphocytes, particularly CD8^+^ T cells that are in relation to favorable survival outcomes (Otegbeye et al., 2021). This suggests that cytotoxic antitumor immune responses participate in modulating tumor development. Checkpoint blockade immunotherapy utilizes monoclonal antibodies to rescue suppressive T cells *via* activating as well as restoring the antitumor activity. Our results demonstrate the aging-related gene model in relation to tumor immunity of rectal cancer.

The tumor microenvironment contains tumor cells, stromal cells, and immune cells as well as extracellular matrix, influencing cancer growth and progression. Aging-related tumor associated fibroblast changes may deteriorate the prognosis of glioblastoma multiforme (Song et al., 2020). Cellular senescence is a critical contributor to the aging process. Here, our data demonstrate that aging HUVECs enhanced proliferation and migration as well as invasion of rectal cancer cells. But AGTR1 knockdown weakened cell senescence and the carcinogenic effect of aging HUVECs. Nevertheless, more experiments will be conducted for verifying the biological implications of aging-related genes in rectal cancer.

## Conclusion

Collectively, this study demonstrates that the aging-related gene signature is in relation to tumor immunity and stromal activation in rectal cancer, which might possess the potential in predicting survival outcomes and immuno- and chemotherapy benefits.

## Data Availability

The original contributions presented in the study are included in the article/Supplementary Material, further inquiries can be directed to the corresponding author.

## Abbreviations

CI, confidence interval; CNA, Somatic copy-number alteration; CMap, Connectivity map; DEGs, differentially expressed genes; DFS, disease-free survival; ESTIMATE, Estimation of Stromal and Immune Cells in Malignant Tumors using Expression Data; EMT, epithelial-mesenchymal transition; FC, fold change; FDR, false discovery rate; GSEA, Gene set enrichment analysis; GO, Gene Ontology; GEO, Gene Expression Omnibus; GDSC, Genomics of Drug Sensitivity in Cancer; HAGR, Human Ageing Genomic Resources; HR, hazard ratio; HLA, human leukocyte antigen; HUVECs, Human umbilical vein endothelial cells; KEGG, Kyoto Encyclopedia of Genes and Genomes; LASSO, least absolute shrinkage and selection operator; NES, nominal enrichment score; OS, overall survival; PFI, progression-free interval; ROC, receiver operator characteristic; RT-qPCR, Real-time quantitative polymerase chain reaction; ssGSEA, single-sample gene-set enrichment analysis; siRNA, small interfering RNA; TCGA, the Cancer Genome Atlas.
