# Supplementary figures and images for: Development of an Aging-Related Gene Signature for Predicting Prognosis, Immunotherapy, and Chemotherapy Benefits in Rectal Cancer
Source: Front Mol Biosci. 2022 Jan 10;8:775700. doi: 10.3389/fmolb.2021.775700 (PMC8784816; doi:10.3389/fmolb.2021.775700)

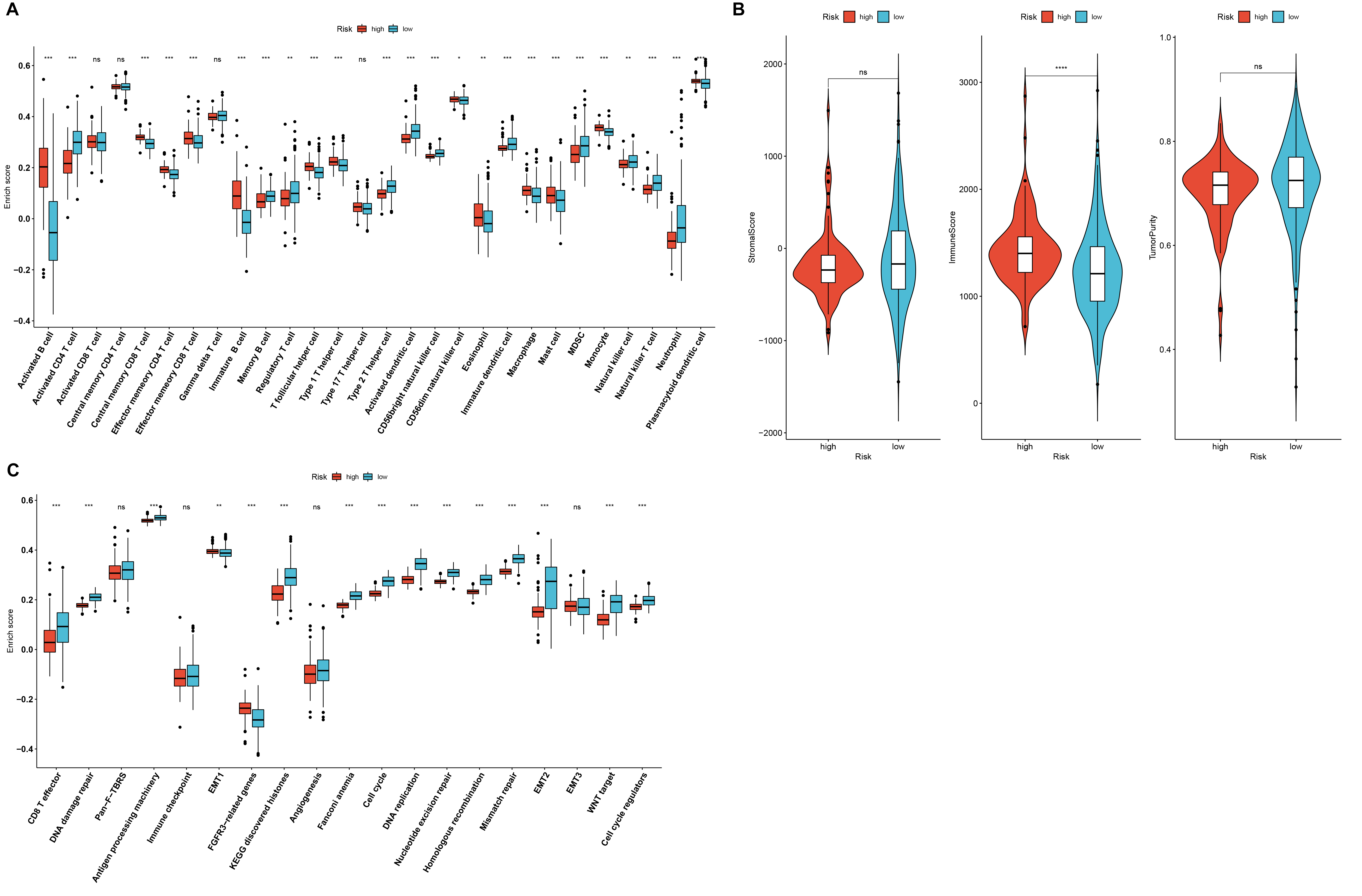

Supplement: Supplementary file 3 [file Image1.TIF]
